# Needle in a Haystack: The Naïve Repertoire as a Source of T Cell Receptors for Adoptive Therapy with Engineered T Cells

**DOI:** 10.3390/ijms21218324

**Published:** 2020-11-06

**Authors:** Elvira D’Ippolito, Karolin I. Wagner, Dirk H Busch

**Affiliations:** 1 Institute for Medical Microbiology, Immunology and Hygiene, Technische Universität München (TUM), 81675 Munich, Germany; elvira.dippolito@tum.de (E.D.); ki.wagner@tum.de (K.I.W.); 2 German Center for Infection Research (DZIF), Partner Site Munich, 81675 Munich, Germany; 3 Focus Group ‘‘Clinical Cell Processing and Purification”, Institute for Advanced Study, Technische Universität München (TUM), 81675 Munich, Germany

**Keywords:** adoptive cell therapy, T cell receptor, naïve repertoire, tumor neoantigens

## Abstract

T cell engineering with antigen-specific T cell receptors (TCRs) has allowed the generation of increasingly specific, reliable, and versatile T cell products with near-physiological features. However, a broad applicability of TCR-based therapies in cancer is still limited by the restricted number of TCRs, often also of suboptimal potency, available for clinical use. In addition, targeting of tumor neoantigens with TCR-engineered T cell therapy moves the field towards a highly personalized treatment, as tumor neoantigens derive from somatic mutations and are extremely patient-specific. Therefore, relevant TCRs have to be de novo identified for each patient and within a narrow time window. The naïve repertoire of healthy donors would represent a reliable source due to its huge diverse TCR repertoire, which theoretically entails T cells for any antigen specificity, including tumor neoantigens. As a challenge, antigen-specific naïve T cells are of extremely low frequency and mostly of low functionality, making the identification of highly functional TCRs finding a “needle in a haystack.” In this review, we present the technological advancements achieved in high-throughput mapping of patient-specific neoantigens and corresponding cognate TCRs and how these platforms can be used to interrogate the naïve repertoire for a fast and efficient identification of rare but therapeutically valuable TCRs for personalized adoptive T cell therapy.

## 1. Introduction

T cells evolved over millions of years to protect the host from infections, through the recognition of target proteins of the pathogen (antigens) via the T cell receptor (TCR) and subsequent T cell activation. Thereby, TCR-activated T cells can clear target cells efficiently with high sensitivity and specificity. This concept has been widely used therapeutically over the last decades to treat infections and tumors by the adoptive transfer of antigen-specific T cells [1]. First convincing proof-of-concept for the therapeutic value of adoptive T cell transfer (ACT) was observed in the context of virus-specific T cells. Infusion of naturally occurring, virus-specific T cells from seropositive donors to patients with compromised immune system conferred protection from reactivation of life-threatening viruses like cytomegalovirus, Epstein-Barr virus, and adenovirus [2,3,4].

In the late 1980s, preclinical studies reported for the first time also the existence of “tumoricidal” T cells within the fraction of tumor infiltrating lymphocytes (TILs). T cells isolated from cancer resections not only showed tumor specificity but also elicited potent antitumor activity when reinfused in tumor-bearing hosts [5,6]. These observations paved the way for the use of ACT in cancer therapy, especially in the form of transfer of autologous, ex vivo expanded TILs [7,8,9]. Unfortunately, after initial success in metastatic melanoma, TIL therapy faced major obstacles that limited its broader applicability. Tumor resection is not always accessible, TILs still fail to expand ex vivo for a fraction of patients and certain tumor types, and maintenance of functionality after expansion cannot be ensured (TILs can become dysfunctional after the extensive in vitro culture) [10,11]. More importantly, in addition to the technical issues related to manufacturing processes, the generated T cell products are usually poorly characterized with respect to antigen specificity and functionality and, therefore, with unpredictable therapeutic efficacy. It was observed that only a small fraction of the highly heterogeneous intratumoral TCR repertoire is able to recognize autologous cancer cells [12,13]. Nevertheless, the restricted set of tumor-specific T cells is in some cases sufficient to mediate complete antitumor efficacy [14,15]. Following this evidence, methods to enrich for these low-abundant tumor-specific T cell clones are currently under development and investigation to generate more tailored TIL products [16,17,18,19]. However, precise definition of specificity and functionality within TILs remains difficult to achieve. Furthermore, prerequisite for a functional TIL-derived product is the presence of pre-existing tumor-specific T cells, which is related to tumor-intrinsic immunogenicity and the degree of exhaustion or deletion of tumor-specific T cells within the tumor microenvironment. The ability to elicit an immune response also varies among patients and tumor types, complicating any prediction of response to TIL therapy.

Overall, adoptive transfer of tumor-specific T cells emerged as the new frontier for cancer treatment, but optimization is still required to have T cell products with robust and predictable therapeutic efficacy. Engineering of T cells with tumor-specific TCRs offers the possibility to generate more reliable and versatile “living drugs,” fundamental features for broader applicability. The transgenic TCR indeed can mediate defined specificity and functionality. Several TCRs specific for tumor-associated antigens (TAA) (MART1, WT1, and NY-ESO among many others) have been already used in clinical trials with modest to high objective responses [20]. However, the overall limited therapeutic efficacy and occurrence of related toxicities point in the direction that much broader repertoire of more functional TCR-engineered T cell products is needed. On the one hand, increasingly sophisticated engineering techniques already allowed to generate engineered T cells with near-physiological functions and likely safer profile. Recently, CRISPR/Cas9 technology was used to introduce a transgenic TCR into the endogenous TCR locus (orthotopic TCR replacement) with consequent benefits on transgenic TCR regulation, expression, and functionality [21,22]. Additional engineering, e.g., knock-out of the remaining endogenous TCR would further increase functionality of transgenic TCRs by eliminating competition for surface expression [23,24]. Presumably, also the risk of undesired toxicities can be minimized by endogenous TCR knockout, as otherwise transgene/endogenous TCR mispairing could generate TCR variants with new antigen specificities not negatively selected in the thymus. Extensive discussion on advantages and recent advancements in orthotopic TCR replacement are beyond the scope of this review and have been discussed elsewhere [25]. On the other side, the TCR still remains the major determinant of T cell functionality. Thus, many efforts are required to identify new and more functional TCRs. In this big challenge for identifying tumor-specific TCRs many questions are still on debate: (i) which is the best tumor-associated antigen to target?, (ii) how could functional TCRs be identified and characterized with high-throughput?, (iii) which is the best source for TCR extraction?, and (iv) how could it be possible to predict cross-reactivity and, thus, off-target toxicities? In this review, we discuss the great potential of the antigen-unexperienced repertoire of healthy donors as source for functional TCRs for ACT, and how this can be integrated with methodologies for high-throughput tumor neoantigen identification and TCR characterization, to finally make possible a patient-specific ACT.

## 2. Identification of Functional Tumor-Specific TCRs for Highly Personalized ACT

In an ideal world, a perfect TCR with therapeutic value should be tumor-specific, highly functional and recognizing a shared target, so that the same TCR could be used to treat a broad range of patients and tumor types. As discussed below, the identification of new promising tumor-specific antigens and the development of TCR functional screenings have been helping to fulfill the first two requirements. Unfortunately, the idea of establishing “off-the-shelf” TCR libraries to treat patients who share the same tumor antigen has become less realistic.

The complexity of TCR target recognition already poses a limitation to the sharing of the same therapeutic TCR. T cells recognize target proteins in the form of short peptides (epitopes) presented by the major histocompatibility complex (MHC) [26], where the epitope is allocated in the MHC binding grove due to interactions between conserved anchor residues of the peptide and amino acids of the MHC. Only some peptides generate a stable pMHCs that are transported to the cell membrane for epitope presentation. MHCs (also named “human leucocyte antigens,” HLAs) are highly polymorphic; thus, each epitope has a different stability for the different HLAs and some epitope-HLA combinations simply do not occur. In addition, only a few HLA alleles are expressed by an individual (only 6 out of hundred variants). This means that i) the same antigen can elicit immune responses through different epitopes according to the HLA context and ii) HLA haplotypes shape the array of presented epitopes of an individual. In conclusion, HLA-restriction of TCR target recognition limits the number of patients who could benefit from the same TCR-based therapy. Furthermore, as summarized in the following paragraph, shared immunogenic antigens unfortunately rarely occur.

Overall, the identification of a functional TCR for successful engineered T cell therapy is a complex equation dictated by the selection of proper immunogenic antigens, the HLA background of the patient, and the intrinsic functionality of individual TCRs.

### 2.1. Clinical Success of TCR-Based ACT According to the Different Tumor-Antigen Classes

TAAs are classified according to their pattern of expression. Differentiation and overexpressed antigens are shared with normal tissues, but usually at lower level. Tumor-specific antigens are, instead, absent in adult cells and comprise, by definition, antigens expressed only in germline cells, during fetal development (carcinoembrionic antigens) or arising from somatic mutations that occur in cancer cells (neoantigens) (Cancer Antigenic Peptide Database). From this classification, it is already reasonable to speculate that selection of tumor-specific antigens, and in particular neoantigens, as targets should provide the most suitable strategy for successful TCR-based immunotherapy. In support of that, 45–67% of melanoma and synovial sarcoma patients clinically benefited from the transfer of TCR-engineered T cell specific for NY-ESO [27,28,29], a TAA expressed at high percentage in several cancer types but only in healthy germline cells [30]. Remarkably, no toxicities were reported. Following the same line, therapeutic use of melanocyte-associated antigen (Melan-1 and gp-100)-specific TCRs showed only low response rates with concomitant on-target/off-tumor side effects due to shared antigen expression with normal melanocytes [31,32]. Despite some success of ACT with NY-ESO TCRs, targeting of tumor-specific antigens still requires caution, especially when affinity-enhanced TCRs are used. Indeed, fatal outcome was reported after the infusion of T cells expressing an affinity-enhanced TCR specific for the melanoma antigen gene (MAGE) A3, but still reactive toward epitopes for MAGE-A6 and MAGE-A12 (one amino acid difference compared to MAGE-A3-derived target epitope). Expression of the highly conserved MAGE superfamily is well-known restricted to reproductive organs [33]. However, an unexpected expression of MAGE-A12 in the brain of a few patients triggered recruitment and activation of the transferred cells with fatal brain damage [34]. Similar off-target/off-tumor toxicities due to loss of specificity with affinity-enhanced TCRs have been reported also in other studies [35,36].

Improved clinical successes in terms of therapeutic efficacy and safety are expected with tumor neoantigen-targeting ACT. As already mentioned, tumor neoantigens derive from somatic mutations and thereby are truly tumor specific. Consequently, the repertoire of endogenous T cell response is not affected by central tolerance and should contain TCRs with higher functionality compared to the repertoire of self TAA-specific TCRs [12,37]. Moreover, on-target/off-target toxicities due to shared antigen expression should be less likely. Obviously, toxicity due to unpredictable reactivity toward homologous peptides cannot be excluded [35] and need to be investigated as part of TCR characterization. Altogether, neoantigen-specific TCRs should have higher functionality and safer therapeutic profile. Remarkably, in first case reports, infusion of a TIL fraction containing high frequency of neoantigen-specific T cells mediated effective antitumor immunotherapy in epithelial cancers, as indicated by complete tumor eradication or prolonged regression [15,38,39]. Longitudinal tracking of the infusion product in the blood circulation of patients showed persistence of these neoantigen-specific T cell clones for up to 17 months [39].

### 2.2. Targeting of Tumor Neoantigens Drives the Next-Generation ACT toward a Personalized Therapy

Over the last years, neoantigen-specific T cell responses have been identified from patient-derived TILs and circulating memory T cells in different tumor types, as described in more detail in the following section. In a few studies, neoantigen-specific TCRs were also identified and re-expressed to validate their specificity and in vitro functionality [40,41,42], thus providing candidate TCRs for engineered T cell therapy. However, intrinsic biological features of tumor neoantigens make it extremely difficult to develop therapeutic strategies with broad applicability. The repertoire of public neoepitopes, i.e., mutated peptides found either in different patients or shared among different tumor types, is very limited. A comprehensive analysis of somatic mutations of 7.748 tumors across 16 different tumor types identified a very few shared neoantigens in tumor-driving oncogenes. Examples are BRAF^V600E^, IDH1^R123H^, and KRAS^G12D/V/R^ mutations found in up to 40% of melanoma/thyroid, glioma, and pancreatic cancers, respectively, but not more than 1–10% of other tumor types [43,44]. In addition, and as already discussed previously, antigen immunogenicity can be driven by different epitopes according to the HLA background of the patient. This further decreases the percentage of patients who could benefit from the use of shared neoantigen-specific TCRs.

Presence of immunogenic neoantigens is extremely hard to predict, as somatic mutations are stochastic events. Tumors with high mutation rates could still lack certain immune-relevant neoantigens and, vice versa, the few mutations in tumors with low mutational burden could be sufficient to elicit tumor responses [12,45,46]. As additional caveat, the burden of neoantigen intratumor heterogeneity could affect the efficacy of TCR therapy. Indeed, identification and targeting of one of a few highly shared neoantigens could not be enough in presence of high fraction of subclonal neoantigens [47,48]. Altogether, each tumor has to be considered as a single entity to map in regards to immunorelevant neoantigens.

Recent advancements in whole exome sequencing (WES) have made it feasible to identify in high-throughput candidate tumor neoantigens via detection of nonsynonymous point mutations (NSPM) in protein-coding genes [27,49,50]. Importantly, only a minor fraction of the neoepitope repertoire seems to be recognized by T cells [51,52], partially due to the still low power of some prediction tools [53]. This is extremely relevant in tumors with high mutational burden, like melanoma and lung cancers [54], where the intrinsic genomic instability would presumably generate larger numbers of new antigens. Therefore, it is crucial to integrate the genomic mapping of neoepitopes with mass spectrometry (MS) and/or prediction tools to analyze, respectively, the tumor ligandome and MHC stability/immunogenicity of peptide candidates (Table 1). Eventually, neoantigens actually presented on tumor cells and with high probability to elicit immune responses are identified. As proof-of-concept, Yadav and colleagues applied this screening approach to mouse tumor models identifying immunogenic neoantigens capable to protect and/or delay tumor growth when administered prophylactically in immune competent mice [55]. Clinical translation followed shortly after, where immunogenic neoantigens were identified and validated also from human tumor resections [14,56,57,58]. Remarkably, neoantigens-specific responses were observed not only in autologous TILs and peripheral blood mononuclear cells (PBMCs) but also in tumor antigen unexperienced T cells of healthy donors [56,58].

Alternative technologies for high-throughput discovery of T cell-recognized antigens have been recently developed. The approach of yeast libraries uses candidate peptides covalently attached to MHC molecules expressed on the surface of yeast for identification of antigen specificity [59]. However, this approach neglects endogenous antigen processing, as well as functional activation. For more physiological technologies, antigen presenting cells (APCs) are first engineered to express antigen libraries, and then cocultured with T cells of unknown specificity. T cell-interacting APCs are detected by the switch-on of reporter genes for T cell activation, e.g., granzyme B sensing systems [60], the expression of fluorescent proteins induced by MHC class I molecules that are engineered with signaling domains [61] or the acquisition of T cell-derived membrane proteins previously labeled with fluorescent markers (physiological process called trogocytosis) [62]. Obviously, antigen libraries are not available for cancer patients as they would be generated after sequencing of individual tumors. Therefore, despite highly innovative, these approaches may be more suitable to decipher orphan TCR-epitopes (e.g., TCRs isolated from TILs) or unravel immunodominant epitopes from pathogens rather than used for the fast identification of immunorelevant patient-specific antigens (Table 1).

Overall, neoantigen-targeting immunotherapy has become a highly personalized approach. Patient-specific neoantigens can be predicted with high-throughput, but the number of candidates still remains high. Therefore, methods for a fast and robust identification of efficiently presented immunogenic neoepitopes and the corresponding functional TCRs are fundamental requirements for progress in this field.

### 2.3. Identification of Functional Neoantigen-Specific TCRs

**Figure 1:**
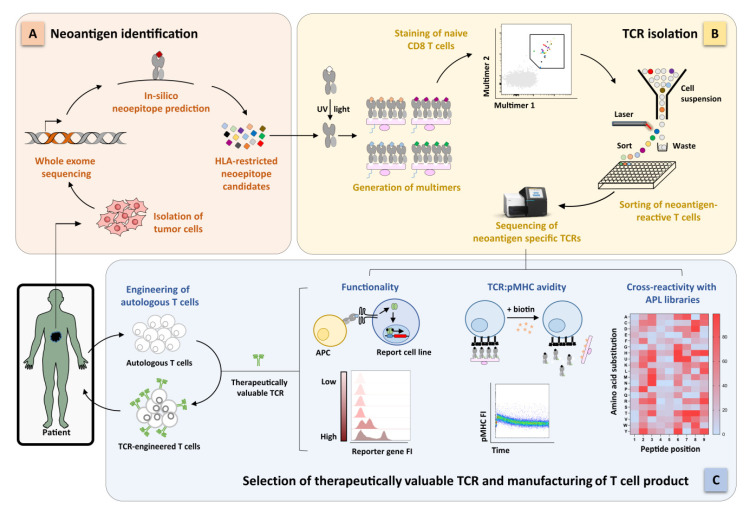
Schematic overview of next-generation personalized adoptive T cell transfer (ACT) targeting patient-derived neoantigens. (**A**) From tumor homogenate, patient-specific neoantigens are mapped via high-throughput whole exome sequencing and further narrowed to candidates using prediction tools for human leucocyte antigens (HLA) binding, proteasomal cleavage, and transport. (**B**) Candidate neoepitopes are rapidly exchanged with conditional major histocompatibility complex (MHC) class I ligands to generate peptide-major histocompatibility complex (pMHC) multimer libraries. The produced DNA-barcoded, fluorophore-labelled multimers are subsequently used to interrogate the naïve repertoire of healthy donors. HLA-matched donor material could be selected from a ready-to-use biobank of naïve T cells. Low-frequency, multimer positive naïve CD8 T cells are sorted and processed for single-cell RNA sequencing, thus retrieving full α/β paired TCRs. (**C**) Candidate T cell receptors (TCRs) are extensively characterized in terms of functionality, structural avidity, and cross-reactivity by the use of, respectively, high-throughput reporter system, TCR:pMHC k_off_-rate and altered peptide ligand (APL) libraries. For TCR:pMHC k_off_-rate, cells are stably labelled with StrepTamer (StrepTagged pMHC multimerized on StrepTactin backbone); the addition of D-biotin disrupts the StrepTamer complex and fluorescently-conjugated pMHC start dissociating, due to the low-affinity of pMHC:TCR interactions. Therapeutically valuable TCRs are finally engineered into patient-derived lymphocytes and infused back into the patient.

pMHC multimer staining has been the gold-standard approach to detect and isolate antigen-specific T cells for the last decades [63]. Conventionally, pMHC multimers consist of monomeric biotinylated pMHCs (MHC class I molecules in vitro refolded with a peptide of interest) multimerized on a fluorophore-conjugated backbone. Our group was one of the first to combine pMHC multimer-guided sorting with single-cell TCR sequencing, thus providing proof-of-principle on how to extract antigen-specific TCRs for immunotherapy [64]. pMHC multimers have not been largely used for the identification of neoantigen-specific TCRs so far. Main reason is that the production of pMHCs is a laborious and time-consuming process, standing against the many candidate neoantigens that would need to be screened. In fact, according to whether WES is combined with mass spectrometry or prediction tools for HLA binding, epitope processing, and immunogenicity, from a handful to hundreds neoantigens can be predicted as immunorelevant [53,65]. The use of mass spectrometry to screen neoantigen ligandomes requires expertise and sophisticated equipment; moreover, many candidates are lost due to limited sensitivity [53]. Thus, the scientific community mostly focuses on the systematic screening of all NSPMs coming from sequencing alone or in combination only with the mentioned prediction tools (Figure 1A).

Two major breakthroughs reinvigorated the attention to the multimer technology. First, the development of methods for fast and flexible generation of many different pMHCs. Here, MHCs are stably loaded with conditional peptides that can be exchanged on command by exposure to UV light [66,67,68,69], increase in temperature [70], or simply incubation with high concentration of the epitope of interest [71,72]. Second, the DNA barcoding of backbones used for pMHC multimerization [73,74,75]. Astonishingly, Bentzen and colleagues combined DNA barcoding with UV peptide exchange to generate a library of 1.031 pMHCs; all peptides were analyzed in parallel, and CD8 T cells with different specificities were detected from size-limited samples [73]. However, information about individual epitope:TCR interactions, particularly sequences of TCRs relevant for immunotherapy, cannot be obtained. Zhang and colleagues brilliantly overcame this limitation through the generation of fluorescently-labeled DNA-barcoded pMHC multimers. In addition to the flexibility of high-scale pMHC production and antigen multiplexing provided by the use of conditional epitopes and DNA tag, the fluorescent label on multimer backbone allowed sorting of the antigen-specific cells, finally available for subsequent TCR sequencing [75] (Figure 1B). Overall, great technological advancements have been made in the pMHC multimer field. Therefore, their role in the identification of neoantigen-specific TCRs will get intensified over the next years.

Besides direct detection via pMHC multimer staining, candidate TCRs for immunotherapy have been found also via approaches involving in vitro stimulation and clonal selection. Briefly, T cells from cancer patients or healthy donors were expanded in vitro, stimulated with peptides of interest and selected according to activation markers related to T-cell functions. These approaches found immediate translation into the tumor neoantigen field, as they adapted faster to the need of testing many different epitopes with unknown specificity and immunogenicity. In addition, using T cell responses as readout, immunogenic epitopes and cognate TCRs may be identified simultaneously. To deal with the huge amount of candidate neoantigens, NSPMs are normally organized either in tandem minigenes or peptide pools. In the first case, each NSPM is encoded by a minigene, where the mutated amino acid is flanked by 12 amino acids of the nonmutated sequence. To increase throughput, 6–24 minigenes can be in-frame linked in a unique tandem minigene, from which RNA is transcribed and transfected in autologous APCs for short-term presentation (intracellular presentation) [40,56,58]. In the second approach, autologous APCs are pulsed with peptide pools composed of up to 24 mutated peptides with a 25 amino acid length (extracellular presentation) [12,41,57]. Reactive T cells from TILs and peripheral blood of patients or healthy donors are finally identified after coincubation with antigen-loaded APCs. Similar to pMHC multimers, also these approaches have been mostly used for the validation of immunogenic neoantigens. Only a few studies deconvoluted tandem minigenes and subsequently identified exact neoepitope:cognate TCR combinations [12,40,41,58].

While this review focuses on the identification of TCRs for individualized ACT in cancer, it is worth to mention that the described methods can be applied also in several other diseases, including infections and transplantations. Indeed, adoptive transfer of virus-specific T cells isolated from seropositive donors via either pMHC multimer-based technology [2] or in vitro selection [3,76] successfully protected immunocompromised patients who received hematopoietic stem cell transplation from life-threatening infections. As additional example, infusion of hepatitis B virus (HBV)-specific T cells could potentially support the therapeutic benefit of liver transplantation in HBV-associated hepatocellular carcinoma [77].

## 3. The Naïve Repertoire of Healthy Donors as Source for TCRs for ACT

**Figure 2:**
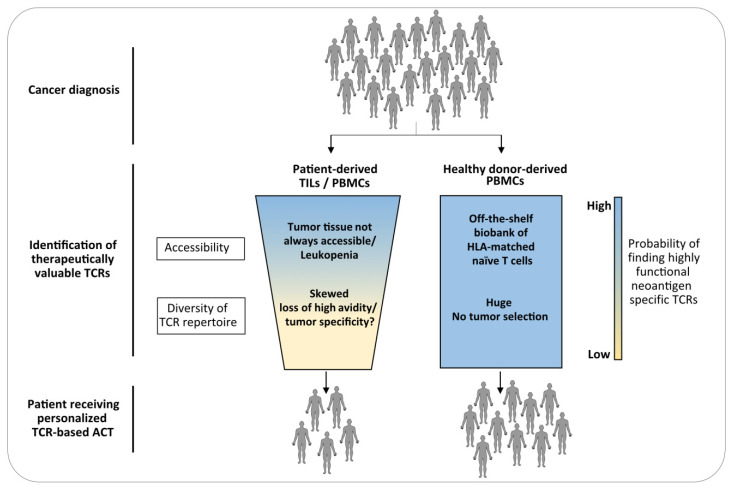
Superiority of healthy donor-derived peripheral blood mononuclear cells (PBMCs) over patient-derived tumor infiltrating lymphocytes (TILs)/PBMCs as a source of therapeutically valuable TCRs. Cancer patients are a limited source for tumor-specific TCR identification, as relevant tumor tissue is not always accessible for TIL isolation and peripheral blood may have low lymphocyte count due to extensive cancer treatment. In addition, the TCR repertoire is often skewed towards lower avidity due to tumor pressure. This results in a reduced probability of finding highly functional neoantigen-specific TCRs, thus limiting access to TRC-based therapies. On the contrary, healthy donor-derived PBMCs are not only an easily accessible source but also offer a broader repertoire to interrogate in respect of any potential specificity, including tumor neoantigens. Higher number of patients would be then eligible for personalized ACT.

Many studies showed that neoantigen-reactive TCRs can be extracted from TILs and circulating memory T cells from patients with different tumor types, e.g., ovarian, hematological malignancies, head and neck squamous cell carcinoma, and other epithelial tumors [12,40,41,78,79,80]. However, as personalized TCR-based ACT requires a rapid identification of therapeutically valuable TCRs within a few months, the use of patient-derived material as a source has many limitations. First, tumors are not always accessible for large resections; smaller resections as well as biopsies should still provide a sufficient number of tumor cells for neoantigen mapping, but not enough TILs for TCR identification. The successful detection of neoantigen-specific T cells from peripheral blood offered a more efficient and noninvasive strategy, though it is still on debate whether circulating memory T cells match TILs in terms of TCR repertoire and, more importantly, functionality. The group of Rosenberg showed that PD-1, activation marker upregulated upon TCR engagement, reliably identify tumor neoantigen-reactive T cells not only within the tumor microenvironment [81] but also in peripheral circulation. Despite present at lower frequency compared to TILs, circulating memory CD8+ and CD4+ PD-1^high^ T cells exhibited marked reactivity toward patient-derived neoantigens [40,82,83]. Remarkably, same reactive TCRs were detected in both circulating T cells and TILs from matched blood and primary tumor [40]. Further validation in other tumor settings is now required. Second, the TCR repertoire of patients is skewed by tumor pressure and high functional TCRs may be lost during tumor progression. It is indeed well-known that tumor cells led to activation-induced cell death of TILs as mechanism of immune evasion [84,85]. Third, patients are often lymphopenic and/or immunocompromised due to extensive pretreatments [86] thereby limiting sample accessibility (Figure 2). 

In contrast, the TCR repertoire of tumor-neoantigen unexperienced healthy donors (referred also as naïve repertoire) is not shaped by tumor selection and has no signature of central tolerance, since neoantigens are distinct from self-antigens. In addition, as naïve T cells should protect individuals from theoretically any kind of pathogens during life, its TCR repertoire is enormously diverse. Therefore, the likelihood to include also highly specific TCRs for any given neoantigen is very high. Finally, peripheral blood from healthy individuals is an easily accessible and almost unlimited source (Figure 2). In a proof-of-principle study by Strønen and colleagues, T cells reactive toward predicted melanoma neoantigens were found in peripheral blood of multiple HLA-matched healthy donors. Interestingly, only around 1% of the neoantigens that induced responses in naïve T cells were capable to stimulate patient-derived TILs [58], highlighting the greater potential of naïve repertoire as source for tumor-specific TCRs.

The use of a donor poses the problem of HLA matching; each patient-derived neoantigen is restricted to a specific HLA allele, which has to be present in the donor. To solve this potential issue, the generation of a biobank of “off-the-shelf” naïve T cells collected from donors with diverse HLA aplotypes can be envisioned, thanks to the easy accessibility of blood from healthy individuals. A set of nine HLA class I alleles (A*01:01, A*02:01, A*03:01, A*11:01, A*24:02, B*07:02, B*08:01, B*35:01, and C*07:02) among the most common human HLAs is already capable to cover 76.5% of the Caucasian population, for which pMHC multimers would be already available [87]. Further implementation of this HLA set would cover most, if not all, of the HLA restrictions.

## 4. Features of the Naïve Repertoire

### 4.1. Generation of the Naïve TCR Repertoire

The ability of the adaptive immune system to respond to a wide variety of pathogens depends on a large repertoire of unique TCRs, the fingerprint of a T cell. The diversity of such repertoire dictates the wideness and efficacy of protection against infections [88,89,90] and could predict outcome and response to therapy in other diseases [91]. Ultimately, estimation of TCR diversity may be useful for immunomonitoring, e.g., after vaccination or therapeutic T cell transfer.

TCR diversity is generated by genomic rearrangement of noncontiguous V and J segments of the TCR α (TCRA) and V, D, and J segments of the TCR β (TCRB) genes in the thymus (γδ TCRs are not discussed in this review). In addition, diversity is further increased by template-independent insertions and deletions of nucleotides at V(D)J junctions, which occur during TCR recombination [92,93] and are responsible for most of the variation among different α/β chains. Among these so-called complementary-determining regions (CDRs), CDR3 regions of both α and β chains contain the highest concentration of diversity, as they directly interact with the antigenic peptide presented by MHC molecules [26]. TCRs with productive rearrangements undergo positive and negative selection, where TCRs with sufficient, but not too high, affinity for any self-pMHC are selected [94]. Elimination of TCRs with high reactivity prevents from the thymic release of potential autoreactive TCRs, whereas, low-affinity interactions with self-pMHC are required for T cell survival in the periphery [95]. At the end, only 3–5% of thymocytes survive and enter the blood circulation [96]. From the 10^20^–10^61^ TCRs that could be potentially generated (TCR clonotypes) [97,98], only 10^6^–10^8^ unique TCRs have been enumerated in adult individuals [99,100,101]. It is important to consider that, despite huge progresses in next-generation sequencing technologies, precise enumeration remains difficult to achieve, e.g., due to limited volume of blood that can be sampled from one person at one time and technical challenging in absolute and unbiased estimation of clonotypes among many others [102]. It will not be surprising if the number of unique TCRs found to compose the immune repertoire will increase along with technology development. In addition, these studies defined clonotypes only according to individual TCRα and TCRβ chains rather than fully α/β paired TCRs; therefore, the number of unique TCRs is already underestimated.

The naïve TCR repertoire is established during the first 20–30 years of an individual life and maintained until elderly due to the high longevity of naïve T cells. The thymic repertoire is transient with different clones produced at different times [103], but generation of new T cells is reduced with age due to thymic involution [104]. Already from the early adulthood, homeostatic proliferation of peripheral T cells becomes the predominant mechanism contributing to the maintenance of the naïve pool [105]. Naïve T cells have considerable longevity. It was observed that the naïve repertoire of elderly individuals is apparently still diverse enough to respond to novel infections, despite a modest reduction in richness [101]. On the contrary, the fitness of individual clones can change later in life. Paradoxically, naïve T cells can increase their ability to survive the longer they stay in the circulation [106]. In line with this evidence, large naïve T cell clones with sizes comparable to memory T cell clones have been detected in aged individuals [101], presumably as result of uneven homeostatic proliferation. The occurrence of these large T cell clones, as a consequence of peripheral fitness selection, can subsequently induce changes in the clone-size distribution [101]. Together with a loss of ability to respond to infections over the years, these perturbations in clone sizes rather than reduced TCR diversity have been recently supposed to contribute to the reduced efficiency of the immune system in elderly.

### 4.2. Clone-Size Distribution

To confer broad protection against new infections, the naïve TCR repertoire should contain as many unique TCRs as possible. But overall, there is limited space for T cells in the body and T cells have to compete with each other for survival stimuli. To reconcile these two requirements, the naïve pool must be composed of small-size clones, meaning that each unique TCR should be expressed by a number of cells just sufficient to quickly react and expand after antigen recognition. Many studies tried to catch the depth of the naïve TCR repertoire with increasingly refined sequencing approaches [100,101,107,108,109]. Despite some confounding factors like repeated mRNA sampling or cell population impurities, most of the studies pointed toward the same conclusion. The naïve repertoire is composed for the vast majority of T cell clones with small sizes, as indicated by the fact that most of the TCRα and TCRβ sequences were normally found only once in the analyzed samples. Accordingly, empirical enumeration of antigen-specific naïve T cells sets the precursor frequency to 1 in 10^6^ naïve cells on average [110]. Due to the abundance of small-size clones, the composition of the naïve repertoire is in addition extremely dynamic. For example, pathological diseases, immunization, and infections may already perturb the repertoire, as the expansion of antigen-specific populations may lead to the disappearance of low-size clones.

A second finding was the observation of a small proportion of large T cell clones, accounting for 1–5% of the total naïve repertoire, with expected frequency greater than 1 in 10^5^ [111]. Thus, individual TCRs are not equally represented. Many mechanisms have been hypothesized to explain the enrichment of certain TCR clonotypes. First, clone-size distribution may be already shaped during TCR generation. The recombination process is not a pure stochastic event but some V(D)J combinations are more likely to occur [112]; TCRs can differ by several order of magnitude in their probability to be recombined [113]. Moreover, different nucleotide triplets can lead to the same amino acid sequence because of redundancy of the genetic code, phenomenon termed convergent recombination [114]. In line with that, first analyses associated a high recombination probability with a higher frequency of the TCR in the naïve population, a mechanism that might explain the observed higher sequence abundance for TCRα but not for the TCRβ repertoire [111]. A second hypothesis suggests that large clones may appear very early in the development of immunity and get stabilized before limitations in survival and rapid division occur. Indeed, most abundant TCR sequences are enriched for sequences without N-insertions [111], a characteristic of TCRs produced prenatally [108]. Nevertheless, it is still an open question whether larger clones have an advantage for selection in recruitment during immune responses.

Overall, the TCR repertoire is composed of an immense, despite limited, number of unique TCRs. TCR diversity is the result of a fine balance between the number of unique TCRs (clonotypes) and the number of T cells expressing the same unique TCR (clone size), where the initial genomic imprinting is shaped by environmental factors occurring during life.

### 4.3. Avidity of the Naïve TCR Repertoire in the Context of Tumor Antigens

In natural T cells, the TCR drives antigen-specificity, functionality, and proliferative fate. The TCR structural avidity, which is defined as the TCR affinity toward the cognate pMHC in presence of CD4/CD8 coreceptors, is a key determinant of T cell functionality [115,116]. Antigen-specific T cell populations are composed of a broad spectrum of high and low-avidity TCRs. It is well-known that high-avidity TCRs dominate the acute phase during primary responses [117] and selectively expand upon recall infection [118,119] being then the main responsible for antigen clearance. Moreover, high-avidity TCRs ensure T cell activation and killing of target cells also at low antigen density. This is extremely important as HLA downregulation in infected or cancer cells, and consequent decrease of epitope density, is a common mechanism of immune evasion [120]. Altogether, high-avidity TCRs are particularly promising candidates for immunotherapy.

The limited clinical success of ACT with TCR-engineered T cells was partially due to inefficient tumor eradication, which would suggest that suboptimal TCRs have been used. This issue primarily depends on the quality of the TCR itself, but it is also linked to the type of tumor antigen selected as target. High-avidity TCRs compose only a minor proportion of a natural polyclonal population specific for foreign antigens [115,121,122]. For self-antigens, the repertoire is further shaped toward lower avidities due to central tolerance, where high-avidity TCRs are eliminated from the repertoire to avoid autoimmunity [123]. Therefore, it is extremely difficult to find high-avidity TCRs from normal TCR repertoires using self-TAA as targets. Attempts to increase TCR avidity by genomic mutation of the TCR sequence usually led to the generation of supraphysiological avidities. Such affinity-enhanced TCRs were problematic for therapeutic application, as above a certain threshold of avidity, functionality, maintenance, and antigen specificity can be lost [35,36]. Moreover, it cannot be excluded that the introduced mutations lead to new specificities. Different might be the situation for carcinoembryonic antigens, as they are only expressed during early development, where the thymus and central tolerance have not been fully established yet. On the contrary, despite unexplored, it is reasonable to speculate that the landscape of TCR avidity against tumor neoantigens should be closer to the repertoire for non-self-antigens, as it is similarly not shaped by negative thymic selection. Experimental proof of this speculation is still lacking and requires further investigation. In support, first evidence showed how clinical responses of TIL therapy have been dominated by high-avidity neoantigen-specific TCRs [15,38,39].

However, in some situations e.g., tumor tissues with high antigen prevalence, the use of high-avidity TCRs may not be optimal. High-avidity TCRs are more susceptible to exhaustion when constantly antigen-triggered or in case of excessive stimulation [124]. Therefore, lower-avidity TCRs, still capable to confer effector functions, may have some advantages as well and recently gained new interest. The potential use of mixtures of low- and high-avidity TCRs to reproduce a natural immune response as new frontiers for T cell immunotherapy has been extensively discussed elsewhere [125].

## 5. Identification of TCRs from the Naïve Repertoire: Challenges and Future Perspectives

### 5.1. Detection of Low-Frequency Antigen-Specific Naïve T Cells

As already discussed above, the naïve repertoire is mainly composed of small-size clones. A consequent drawback is the extremely low frequency of antigen-specific naive T cells, estimated in the range of 0.6–3.6 × 10^−6^ naïve CD8 T cells [110] (frequencies are higher than 10^−5^ for memory T cells). As proof-of-principle, it was shown that it is possible to identify such rare neoantigen-specific naïve T cells from limited amount of blood when an in vitro expansion step is involved [58]. However, the approach itself is laborious and can be further biased by the in vitro cultures. In addition, the probability to miss reactive TCRs due to detection limits rather than absence of specific T cells is relatively high.

Therefore, it would be more efficient a direct ex vivo detection, even if extremely challenging. pMHC multimers currently represent the only technology to directly identify antigen-specific T cells (without the need of activation). T cell activation-related markers, e.g., PD-1 and 4-1BB, successfully used for the enrichment of neoantigen-specific T cells from patient-derived material, are not useful in tumor-free healthy donors. Notably, Alanio and colleagues showed that, despite the detection limit of pMHC multimer staining (around 10^−5^), a pre-enrichment step is sufficient, but also mandatory, to reliably detect antigen-specific naïve T cells [110]. Therefore, detection of rare neoantigen-specific naïve T cells would become a “numbers game” possible to overpower with the use of large T cell sources, e.g., CD3 + leukapheresis, which provides approximately 10^9^ T lymphocytes.

### 5.2. High Abundance of Low Avidity TCRs

The naïve TCR repertoire is mainly composed of low functional—low avidity—TCRs [126]. Therefore, each isolated TCR needs extensive characterization before any clinical application, which is still a time- and cost-consuming step. This includes re-expression in primary T cells for at least the validation of TCR specificity (e.g., via pMHC multimer staining), evaluation of its functionality in vitro and in vivo and, possibly, screening of potential cross-reactivity. Readouts for determining TCR functionality during the process of antigen-specific T cell isolation are highly required and are still lacking in the field. Unnecessary validation of nonfunctional TCRs would be then avoided or at least minimized.

pMHC multimers have been widely used in the last decades for detection and sorting of antigen-specific T cells. Major advantage is the direct isolation of target cells ex vivo, e.g., T cell phenotype can be analyzed in parallel, unlike approaches involving in vitro expansion where phenotype is biased by the culture conditions. pMHC multimer technologies have dramatically evolved over the last 5 years, reaching high-throughput applications for identification of therapeutically valuable TCRs. Thanks to these achievements, it is now possible to generate large-scale pMHC libraries in relative short time, facilitating the detection of T cells with many specificities simultaneously. Moreover, multimerization of pMHC libraries on fluorophore-conjugated DNA-barcoded backbones allows to decipher individual epitope:TCR interactions from bulk analyses of hundreds of epitopes and T cells [75]. Up to date, the highest throughput was achieved by combining multiplexed pMHC multimer staining with single-cell sorting and subsequent single-cell TCR/barcode amplification. cDNA libraries were finally sequenced on next-generation sequencing platforms [75]. Interestingly, largely improved single-cell RNA sequencing (scRNA-seq) technologies [127,128] can be implemented to replace the more laborious process of TCR sequencing just described before. Considering (i) the limited number of immunodominant neoantigens expected to be identified from a single patient [56,58] and (ii) the low-precursor frequency of antigen-specific T cells in healthy donors [110], it is highly probable that the sortable pMHC multimer positive cells will be far below the maximum capacity of a single scRNA-seq run. Consequently, more than one donor may be pooled and analyzed simultaneously. Individual donors can be efficiently demultiplexed according to donor-specific single nucleotide polymorphisms [129,130]. However, a major caveat still exists, which is that TCRs with low and high functionality cannot be discriminated (Table 1). The loading of many pMHCs on a same backbone was appositely designed to stabilize also low avidity TCR:pMHC interactions, which in a physiological T cell:APC crosstalk would not trigger any T cell activation. In addition, intensity of multimer staining does not correlate with functionality. Establishment of functional screening that could be combined with pMHC multimer platforms are indeed urgently needed. TCR avidity could represent a reliable parameter to implement into the workflow of TCR isolation, as it correlates with T cell functionality and it is independent from T cell phenotype [123,131].

Alternatively to direct detection, other approaches used T cell responses after peptide stimulation, either using crude peptides or pulsed APC, to identify neoantigen-specific TCRs [12,40,41,56,57,58]. By involving a T-cell functional readout, these methods may have some interesting advantages. But the disadvantages are probably exceeding for several reasons. First, donor T cells require a period of in vitro expansion before being challenged with peptide stimulation; this alters T cell phenotype, and consequently responsiveness to stimuli, and may induce loss of T cell clones according to individual T cell fitness to the in vitro culture (Table 1). Naïve T cells are the most robust T cell subset in terms of fitness [132,133,134] but still a more independence from culture-related biases needs to be investigated in detail. Second, T cells are stimulated via a “single peptide dose.” Thus, low and high avidity TCRs still cannot be distinguished properly. Altogether, caution must be taken in interpreting these T cell responses more than a digital response. Besides technical confounders, another limitation is that in vitro culture and expansion-based technologies are very labor intensive and unlikely to be transferred to real high-throughput conditions.

### 5.3. Preclinical Validation of TCR Functionality

Neoantigen-specific candidate TCRs require in-depth preclinical characterization regarding specificity and efficacy before any clinical application. This is extremely relevant for TCRs isolated from the naïve repertoire due to the high abundance of low-functional TCRs. These features are normally determined by in vitro assays where T cell functions, e.g., cytokines release, upregulation of activation markers, and cytotoxicity are evaluated in TCR-engineered primary T cells after peptide stimulation or coculture with target cells. If patient-derived tumor cells, which endogenously present the target peptide, are not available, artificial APCs can be used as alternative [135]. However, these assays are still laborious and time intensive, besides the limitation that the readout strongly depends on the T cell phenotype during culture. On the contrary, T cell reporter systems could provide higher throughput (Figure 1C). Rosskopf and colleagues described a triple parameter reporter model based on a T cell lymphoma-derived cell line (Jurkat cells) devoid of endogenous TCR and engineered with three different fluorescent protein-encoding genes, each located downstream a promoter which is activated by TCR-triggered transcription factors [136]. Thus, antigen-reactive TCRs can be easily identified via fast recording of reporter fluorescences. Obviously, the model does not perfectly mirror the physiological T cell signaling and, therefore, is not reliable in evaluating subtle differences in TCR functionality. However, it does still represent a more suitable platform for high-throughput screening than engineered primary T cells. This holds true especially in the case where candidate TCRs have been identified via pMHC multimer technology, as the proportion of very low-avidity TCRs would be relatively high. Similar reporter systems are indeed urgently needed in the field.

Complementary, if not alternative, approach to functional T cell assays is the measurements of TCR structural avidity (i.e., k_D_ = k_on-rate_/k_off-rate_ of the antigen receptor–ligand interaction). This parameter can be accurately quantified by biophysical assay such as surface plasmon resonance, which, however, requires recombinant production of both the TCR and cognate pMHC [137]. TCR:pMHC k_off_-rate determines most of the TCR structural avidity and has the potential to be a robust biomarker for TCR potency, as it correlates with T cell functionality and it is independent from the activation state of T cells [123,131]. Fast flow cytometry-based assays for TCR k_off_-rate measurements are currently available [138,139] (Figure 1C).

In vivo characterization and functional assessment of tumor-specific TCRs proves rather difficult as mice models are not easily available for the vast set of targets and HLA restrictions. Mostly, ACT trials relied on preclinical studies using HLA class I transgenic mice [140,141] or immunodeficient mice engrafted with human tumor cell lines that are HLA-matched with the infused effector T cells [142,143,144]. However, both models are available for a very limited number of HLA alleles. In addition, xenograft models also lack the expression of the human target antigen on normal host tissue, thus excluding evaluation of on-target/off-tumor toxicity [145,146,147,148]. The patient specificity of target neoantigens builds a big obstacle as it precludes the use of any already established xenograft models. Patient-derived organoid cultures have been of great utility to study tumor genetics, development of pathogenesis, and drug toxicity (reviewed in [149,150]). Recently, such organoids have been successfully used for artificial reconstitution of the tumor microenvironment [151] and to study T cell-mediated in vivo cytotoxicity [152]. Worth of investigation could be also the use of patient-derived xenograft (PDX), extensively used in preclinical testing of synthetic antitumor drugs for tailored precision medicine [153,154]. A first line of applicability was described by the Biernacki group, who studied the antitumor efficacy of a neoantigen-specific T cell clone in a PDX model of acute myeloid leukemia [155]. Clearly, the implantation of a solid tumors remains a bigger challenge.

### 5.4. Prediction of TCR Cross-Reactivity

TCR crystallography revealed the ability of a TCR to cross-react to a related peptide. Although clonal selection during T cell development imposes that a T cell is specific for a single peptide-MHC complex, TCR cross-reactivity is instead a natural occurrence. Estimates of CD8 T cell targets quickly exceed 10^18^ peptides by simply accounting for combination of the 20 naturally occurring amino acids and neglecting post-translational modifications [156,157]. Thus, TCR binding degeneracy is required with regards to the limited number of immune cells in a human body. Comprehensive experimental and mathematical analyses estimate that a single TCR can recognize more than a million different pMHCs [158,159], which is highly advantageous in responding to pathogens that otherwise would escape immune recognition by single mutations. However, T cell cross-reactivity is also implicated in the development of autoimmune diseases, when a certain degree of sharing between a pathogen-derived epitope and a self-epitope occurs [160,161,162]. The autoimmune disease of multiple sclerosis builds a common example of TCR cross-reactivity between an Epstein–Barr virus epitope and myelin basic protein [163]. In the context of personalized TCR-based ACT, the isolation of therapeutically relevant TCRs from a source different than the patient, i.e., healthy donors may raise the concern of cross-reactivity as the TCR was naturally selected to tolerate the donor but not the patient self-proteome. Thus, extensive assessment of potential cross-reactivity would be required before clinical application.

Besides a number of in silico prediction tools for T cell cross-recognition [164,165], experimental validation remains crucial. For obvious reasons, the cross-reactivity landscape against any tissue type and of all developmental stages is not feasible. A common approach is to design libraries of altered peptide ligands (APLs) where all possible aberrant target peptides could be simulated by alanine scan, single amino acid substitution, or combinatorial strategy (all 20 naturally occurring amino acids are replaced at any position in the peptide sequence) [159,166] (Figure 1C). Peptide libraries expressed by MHC displaying yeast cells can bind tetramerized TCRs of interest and cross-reactive peptides can be identified via sequencing of the bound pMHC-yeast cell [167]. As alternative, T cell-function readouts, e.g., cytokine and degranulation assays have been used to investigate cross-reactivity in TCR-engineered T cells stimulated with APL library pools. Obviously, any high-throughput approach previously described for antigen discovery and validation could find application here. This would be the case for the already described use of tandem minigene [40,56,58], trogocytosis-based assay [62], and APC-reporting systems [60,61]. pMHC multimer technology has also been applied for cross-reactivity assessment. Neoantigen-specific TCRs can be easily evaluated for cross-reactivity toward the corresponding nonmutated epitope already during the step of TCR isolation by the use of pMHC multimers labeled with different fluorophores [75]. Along the idea of multimer recognition, Bentzen and colleagues established a fast workflow, called as one-pot system, to assess a TCR cross-reactivity landscape by combining DNA-barcoded pMHCs with APL libraries [168]. The TCR cross-reactivity fingerprint was first defined on the basis of TCR recognition to pMHC libraries containing single amino acid substitution APLs. The identified recognition motif has been finally used to scan the human proteome for potential cross-recognized peptides having a partial degree of homology.

## 6. Conclusions

Targeting of tumor neoantigens has emerged as the new frontier for a more efficient and safer ACT, including TCR-based therapies. This increase in precision has been gained, however, at the expensive of a more generic therapy, as tumor neoantigens are extremely patient-specific. Only a small fraction of patients shares public neoantigens in similar HLA restrictions, for which treatments with “off-the-shelf” TCRs would still represent a valid option to pursue. For the remaining majority, functional and safe TCRs have to be de novo identified within a narrow temporal window after the mapping of patient-derived neoantigens, in order to manufacture autologous TCR-engineered T cells in time for therapy. By combining whole exome sequencing and DNA-barcoded pMHC multimer libraries, it is nowadays possible to rapidly get access to hundreds of candidate immunogenic neoantigens and cognate TCRs. Blood from HLA-matched healthy donors would represent the best source for TCR isolation for two main reasons. First, blood is an accessible source that could be stored in ready-to-use biobanks of HLA-matched naïve T cells. Second, the TCR repertoire is extremely diverse and not skewed toward low affinities by tumor pressure or central tolerance. Thus, the probability to find therapeutically relevant TCRs is relatively high. The challenge caused by extremely low frequencies of antigen-specific naïve T cells could be overcome using large amounts of input cells and including a pre-enrichment step. On the contrary, the abundance of low-avidity TCRs remains a major limitation, as functional screenings to implement during the isolation process are still lacking. Therefore, isolated TCRs still require extensive preclinical screening. The existing models for high-throughput in vitro TCR characterization and prediction/experimental validation of cross-reactivity are promising but still at their infancy, thus requiring extensive upcoming research. Similarly, major efforts must be invested in the generation of suitable in vivo models for the evaluation of on target functionality as well as “on and off” target toxicities.

## Figures and Tables

**Table 1:** Advantages and disadvantages of high-throughput methods for neoantigen identification and T cell receptor (TCR) isolation/characterization.

Strategies	Advantages	Disadvantages	Suitable for Personalized ACT
**Identification of Neoantigens**			
Whole exome sequencing	Fast and high-throughput	No information on epitope presentation and immunogenicity	Yes
Mass cytometry of HLA-ligandome	Identify naturally HLA-presented antigens	Require sophisticated equipment Low sensitivity	No
In silico peptide prediction	Easily accessible	Prediction tools are not always accurate, in particular for HLAs with low frequency	Yes
pMHC yeast library	Precise neoantigen target and direct TCR identification	Neglects endogenous antigen processing and lacks functional readout	No
Engineered APCs	Physiological neoantigen presentation. Functional readout	Dependency on predefined antigen library	No
Trogocytosis	Simultaneous identification of TCR and neoantigen	Dependency on predefined antigen library Lack of functional readout	No
**Identification of Neoantigens-Specific TCRs**			
pMHC multimer libraries	High versatility and throughput	Lack of functional readout (pMHC multimer staining does not correlate with T cell functionality)	Yes
Autologous T-cell function assays	No HLA restrictions	Highly dependent on phenotypic status at the time of isolation Potential bias by the in vitro culture	No
**Functional Characterization of TCRs**			
Primary T cells	Physiological T cell signaling, identical to infusion product	Variability in cellular phenotype	No
Reporter cell lines	High throughput, standardized TCR validation	Less physiological, less sensitive for subtle differences between TCRs	Yes

## Abbreviations

ACT	Adoptive T cell therapy
APC	Antigen presenting cells
APL	Altered peptide ligand
HLA	Human leucocyte antigen
CDR	Complementary-determining region
NSPM	Nonsynonymous point mutations
MHC	Major histocompatibility complex
MS PBMCs	Mass spectrometry peripheral blood mononuclear cells
pMHC	Peptide-major histocompatibility complex
TAA	Tumor-associated antigen
TCR	T cell receptor
TILs	Tumor infiltrating lymphocytes
WES	Whole exome sequencing
